# Mendelian Randomization Analysis of the Effect of Maternal Homocysteine During Pregnancy, as Represented by Maternal *MTHFR C677T* Genotype, on Birth Weight

**DOI:** 10.2188/jea.JE20120219

**Published:** 2013-09-05

**Authors:** Hye Ah Lee, Eun Ae Park, Su Jin Cho, Hae Soon Kim, Young Ju Kim, Hwayoung Lee, Hye Sun Gwak, Ki Nam Kim, Namsoo Chang, Eun Hee Ha, Hyesook Park

**Affiliations:** 1 Department of Preventive Medicine, School of Medicine, Ewha Womans University, Seoul, Korea; 2 Department of Pediatrics, School of Medicine, Ewha Womans University, Seoul, Korea; 3 Department of Obstetrics and Gynecology, School of Medicine, Ewha Womans University, Seoul, Korea; 4 Department of Anatomy, School of Medicine, Ewha Womans University, Seoul, Korea; 5 Colleage of Pharmacy, Ewha Womans University, Seoul, Korea; 6 Department of Food and Nutrition, Daejeon University, Daejeon, Korea; 7 Department of Nutritional Science and Food Management, College of Health Sciences, Ewha Womans University, Seoul, Korea

**Keywords:** birth weight, homocysteine, intrauterine environment, Mendelian randomization, methylenetetrahydrofolate reductase

## Abstract

**Background:**

We used Mendelian randomization analysis to investigate the causal relationship between maternal homocysteine level, as represented by maternal methylenetetrahydrofolate reductase (*MTHFR*) *C677T* genotype, with the birth weight of offspring.

**Methods:**

We recruited women at 24 to 28 weeks’ gestation who visited Ewha Womans University Hospital for prenatal care during the period from August 2001 to December 2003. A total of 473 newborns with a gestational age of at least 37 weeks were analyzed in this study. We excluded twin births and children of women with a history of gestational diabetes, gestational hypertension, or chronic renal disease. The association of maternal homocysteine concentration with the birth weight of infants was analyzed using 2-stage regression.

**Results:**

*MTHFR C677T* genotype showed a dose–response association with homocysteine concentration for each additional *T* allele (*P*_trend_ < 0.01). Birth weight decreased from 120 to 130 grams as maternal homocysteine level increased, while controlling for confounding factors; however, the association was of marginal significance (*P* = 0.06).

**Conclusions:**

Our results suggest an adverse relationship between maternal homocysteine level and birth weight. A reduction in homocysteine levels might positively affect birth outcomes.

## INTRODUCTION

The extent of intrauterine growth can determine lifelong health and the health of future generations. During recent decades, a number of studies have confirmed Barker’s hypothesis regarding the fetal origin of adult disease.^1^ The intrauterine environment responds to factors such as abnormal metabolic exposures induced by genetic variants, behavioral features, and environmental factors. In addition to these risk factors, homocysteine concentration was reported to be positively associated with the risk of pregnancy complications^2^ and low birth weight.^3^ In general, homocysteine concentration remains low during pregnancy.^2^ However, abnormal homocysteine levels in the intrauterine environment could induce adverse birth outcomes such as pregnancy loss,^4^^,^^5^ placental abruption,^5^ and intrauterine growth retardation.^3^

Homocysteine metabolism is affected by many factors, including age, gender, current diet, and methylenetetrahydrofolate reductase (*MTHFR*) genotype.^2^ *MTHFR* is a critical enzyme required for the synthesis of 5-methyltetrahydrofolate, an active form of folic acid in blood. It is involved in converting homocysteine to methionine,^6^ and a known polymorphism of *MTHFR* is a transition of *C* to *T* at nucleotide 677 of the *MTHFR* gene, which is a cause of reduced *MTHFR* activity^7^ and hyperhomocysteinemia.^8^ Moreover, the Hordaland Homocysteine Study reported a trend toward restricted intrauterine growth as the number of *T* alleles increased.^9^ In addition, previous studies reported that women homozygous for *C677T* in *MTHFR* had an increased risk of preeclampsia and placental vasculopathy.^2^^,^^3^ Although previous studies showed that *MTHFR C677T* mutation could lead to hyperhomocysteinemia and retardation of intrauterine growth, there is no direct evidence that variation in maternal homocysteine due to *MTHFR* genotype affects offspring growth in utero. One approach to investigate causal inference, Mendelian randomization, is an application of instrumental variable (IV) analysis that uses genetic instruments, due to the principal of independent assortment of an allele during meiosis. The use of Mendelian randomization in an observational study was introduced by Katan.^10^ In the present study, we use maternal genotype as an unconfounded and unbiased instrument for homocysteine level in maternal blood. The application of IV analysis using maternal genetic variants allows us to estimate intergenerational effects.

We used IV analysis to investigate the causal relationship between homocysteine level in the intrauterine environment, as determined by maternal genotype, and offspring birth weight (a maker of intrauterine growth).

## METHODS

### Study subjects

We recruited women between 24 and 28 weeks’ gestation who visited Ewha Womans University Hospital in Seoul, Republic of Korea for prenatal care during the period from August 2001 to December 2003. All participants gave their informed consent for study participation. Our analysis of deliveries during the period from October 2001 to February 2004 (recruiting date, from August 2001 to December 2003) showed that 2481 women gave birth in Ewha Womans University Hospital. About 30% of them were included in this study (*n* = 720). For all participants, venous blood was collected from the antecubital vein during the morning after an overnight fast, and all participants responded to structured questionnaires. We excluded women who had twin births (*n* = 30), gestational diabetes (*n* = 23), hypertension, or chronic renal disease (*n* = 13), those with no data on homocysteine or folate level due to inadequate blood sampling (*n* = 92), and those with incomplete questionnaires (*n* = 89). Therefore, a total of 473 newborns were analyzed for this study (242 boys; 231 girls). There was no difference between participants and excluded subjects in the frequencies of the *CC*, *CT*, and *TT* genotypes (34.2%, 49.7%, and 16.1% for participants vs 32.3%, 49.6%, and 18.2% for excluded subjects, respectively). This study was approved by the Institutional Review Board of Ewha Womans University Hospital.

### Genotyping

Extraction of DNA from fasting venous blood was done using a QIAmp DNA blood kit (Qiagen, Hilden, Germany) according to the protocol recommend by the manufacturer, and samples were stored at −20°C until analysis. *MTHFR C677T* genotypes were identified by polymerase chain reaction (PCR) amplification and digestion with *Hinf1*. The PCR product resulted in synthesis of 198 bp fragments, which contain a *C* to *T* substitution at nucleotide 677 of the *MTHFR* gene. The fragments were cut into 175 and 23 bp fragments by the derived T allele and were visualized on 2.5% polyacrylamide gel with ethidium bromide staining after electrophoresis.

### Laboratory measurements

High-performance liquid chromatography was used to measure homocysteine. Briefly, we mixed 100 µL of plasma with 10 µL of 10% tri-n-butylphosphine and stored the sample for 30 min at 4°C. Deproteinization was performed by adding 100 µL of 10% trichloroacetic acid, followed by centrifugation for 5 minutes at 3000 g. The supernatant was mixed with 250 µL of 0.125 M borate buffer and 100 µL of ammonium 7-fluorobenzo-2-oxa-1,3-diazole-4-sulfonate acid. After incubation for 1 hour at 60°C, the mixture was filtered with a 0.45-µm syringe filter (HV type, Whatman). Fluorescence detection was performed (excitation 385 nm, emission 515 nm) using an Xterra RP_18_ column. Folate was measured by a radioimmunoassay kit (Diagnostic Products Corporation, Los Angeles, CA, USA).

### Clinical data

Birth weight and length were measured in the delivery room by trained nurses using digital scales. Gestational age was calculated based on the maternal report of the date of last menstrual period and ultrasound measurement performed by an obstetrician.

### Statistical analysis

Data analysis was conducted using the statistical package STATA (version 11.0). In descriptive analysis, continuous variables were presented as mean with SD and categorical variables as frequencies. Genotype was analyzed both as a categorical variable based on number of *T* alleles (in the codominant model) and as a continuous variable (in the additive model). We used a general linear model and the χ^2^ test to assess associations of confounding variables with the different *MTHFR* genotypes.

Mendelian randomization analysis was done using the “ivreg” command in STATA. Two-stage least-squares estimates were used to fit the IV analysis. Maternal *MTHFR C677T* genotype was used as an IV to estimate the association between intrauterine homocysteine environment and the birth weight of offspring. Gestational age, the sex of the offspring, maternal age, and folate concentration were analyzed as confounding variables. We used the Durbin form of the Durbin–Wu–Hausman statistic to compare estimates of the effects of homocysteine on birth weight obtained from ordinary least-squares linear regression and IV analysis.^11^

## RESULTS

Among the 473 subjects, 242 were male (51.2%), and mean birth weight was 3299 grams. The genotype frequencies of *CC*, *CT*, and *TT* in the included (34.2%, 49.7%, and 16.1%, respectively) and excluded (32.3%, 49.6%, and 18.2%, respectively) subjects were similar and were in Hardy–Weinberg equilibrium (*P* > 0.05). The mean homocysteine concentration of the subjects was 5.76 µmol/L and did not significantly differ from that of excluded subjects (6.03 ± 1.64 µmol/L, *P* = 0.33) (Table 1).

**Table 1.** Basic characteristics of the study subjects

Factors	Study subjects (*n* = 473)
Maternal age (years)	30.99 (±3.56)
Maternal pre-pregnancy weight (kg, *n* = 393)^a^	53.89 (±7.58)
Maternal weight gain during pregnancy (kg, *n* = 388)^a^	12.88 (±4.64)
Homocysteine concentration (µmol/L)	5.76 (±1.53)
Folate concentration (ng/mL)	6.79 (±1.93)
*MTHFR C677T* genotype	
*CC*	162 (34.25%)
*CT*	235 (49.68%)
*TT*	76 (16.07%)
Gestational age (weeks)	39.4 (±1.1)
Offspring sex (male)	242 (51.16%)
Offspring birth weight (g)	3299 (±376)
Offspring length at birth (cm)	49.47 (±1.80)

Numeric values represent mean (SD), and categorical variable represent *n* (%).

^a^The numbers of subjects differ due to missing data.

Homocysteine concentration was significantly associated with *MTHFR C677T* genotype and showed a dose–response association for each additional *T* allele: namely, *CC* (5.47 µmol/L), *CT* (5.75 µmol/L), and *TT* (6.48 µmol/L) (*P* for trend < 0.01; Figure). In contrast, no *MTHFR C677T* genotype was associated with any potential confounding factor (Table 2).

**Figure.**
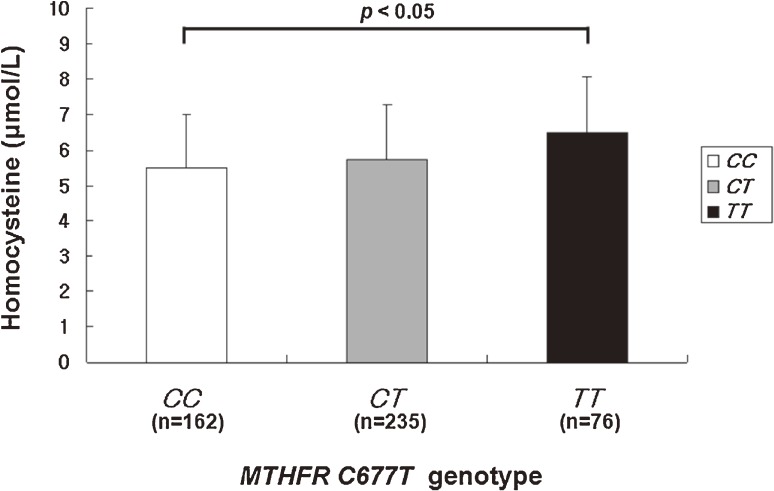
Homocysteine concentration (µmol/L) according to *MTHFR C677T* genotype

**Table 2.** Association of maternal *MTHFR C677T* genotype with potential confounding factors

Factors	Maternal *MTHFR C677T* genotype			*P* value^a^

	*CC* (*n* = 162)	*CT* (*n* = 235)	*TT* (*n* = 76)	
Maternal age (years)	31.02 (3.37)	30.98 (3.63)	30.95 (3.76)	0.99
Folate concentration (ng/mL)	6.91 (1.91)	6.97 (1.97)	6.03 (1.84)	0.23
Gestational age (weeks)	39.5 (1.1)	39.3 (1.1)	39.4 (1.0)	0.35
Offspring sex (male)	84 (51.85%)	123 (52.34%)	35 (46.05%)	0.62

^a^The *P* value of parametric analysis was estimated using a generalized linear model.

Birth weight significantly differed in relation to *MTHFR C677T* genotype (*CC*: 3.35 ± 0.03 kg; *CT*: 3.29 ± 0.02 kg; *TT*: 3.23 ± 0.04 kg; *P* value = 0.04) after controlling for gestational age and sex. In particular, adjusted mean birth weight for the *TT* allele was lower than that for the *CC* allele (*P* = 0.04, after Tukey–Kramer adjustment for multiple comparisons). However, homocysteine level was not significantly associated with birth weight, even after adjustment for confounding variables (data not shown).

Table 3 shows the results of IV analysis of maternal intrauterine homocysteine level in relation to growth of the offspring. Birth weight decreased by 120 to 130 grams as maternal homocysteine level increased (codominant model: β = −120.90, 95% CI −246.88 to 5.08; additive model: β = −130.28, 95% CI −266.68 to 6.13) after adjustment for potential confounding variables, although this association was only marginally significant (*P* = 0.06). Ordinary least-squares regression analysis showed a clear distinction in the direction of the associations, and the results of IV analysis and the Hausman test were statistically significant (*P* < 0.05). This suggests that, as compared with the ordinary least-squares method, IV analysis is more suitable for analyzing the relationship of maternal homocysteine and birth weight (data not shown).

**Table 3.** Effect of homocysteine on birth weight, as determined by Mendelian randomization analysis using maternal *MTHFR* genotype as the instrumental variable

Model	Codominant model		Additive model	
	
	Coefficient β (95% CI)	*P*	Coefficient β (95% CI)	*P*
Simple model	−104.7 (−206.15, −3.25)	0.04	−123.95 (−240.77, −7.12)	0.04
Multiple model				
Model 1	−91.95 (−187.90, 4.01)	0.06	−106.42 (−216.08, 3.25)	0.06
Model 2	−120.90 (−246.88, 5.08)	0.06	−130.28 (−266.68, 6.13)	0.06

Model 1 adjusted for gestational age and offspring sex.

Model 2 adjusted for above covariates plus maternal age and folate concentration.

## DISCUSSION

We found a strong association between *MTHFR C677T* genotype and homocysteine level. There was a dose–response association between homocysteine and homozygosity for *MTHFR C677T* (*TT*), ie, homocysteine level significantly increased with the number of *T* alleles (*P* for trend <0.01). Furthermore, the findings of our advanced statistical approach suggest that maternal homocysteine concentration during pregnancy has an adverse effect on the birth weight of offspring, although the result was of marginal significance (*P* = 0.06), perhaps due to limited statistical power or the small sample size. To our knowledge, no other study has used an advanced statistical approach to investigate the association of intrauterine homocysteine exposure, as determined by maternal *MTHFR* genotype, with offspring birth weight.

As mentioned above, the use of maternal gene polymorphism as an instrumental variable in our analysis allowed us to use *MTHFR* genotype to estimate the causal effect of variation in maternal homocysteine. The characteristics of Mendelian randomization permit genetic variants to be investigated in an observational study. Because such studies have properties similar to those of intention-to-treat analyses in randomized controlled trials,^12^ the problems of observational studies, eg, reverse causality and bias due to confounding factors, are avoided.^13^ In addition, genetic variants have larger effects on intermediate features than on the disease itself.^14^ The *MTHFR C677T* polymorphism is already established as an instrument: a study using Mendelian randomization reported that homocysteine level, as determined by *MTHFR C677T* gene variant, was associated with an increased risk for stroke.^15^

Homocysteine is normally found in blood, and it has been suggested that increased levels impair endothelial vasomotor function and facilitate platelet production, thereby damaging arteries and blood flow.^3^ A causal role for homocysteine in the pathogenesis of metabolic disorders has been suggested in hypertension, congenital heart disease, and cardiovascular disease.^3^^,^^8^^,^^15^ In addition, many previous studies have reported that homocysteine was associated with adverse effects on pregnancy and birth outcomes^2^^,^^5^^,^^16^; however, opinions differ in relation to causality. Regarding the genetic effect on homocysteine concentration, a transition of *C* to *T* at nucleotide 677 of the *MTHFR* gene was shown to cause hyperhomocysteinemia, which could inhibit *MTHFR* enzyme activity. Moreover, this inhibition of *MTHFR* enzyme activity might be amplified by folate deficiency.^2^ As in previous studies, we found that homocysteine negatively correlated with folate concentration in blood (*r* = −0.33, *P* value <0.0001) and that homocysteine level was inversely associated with offspring birth weight, even after controlling for folate concentration in blood and other potential confounders. Studies reported that babies with *MTHFR* mutation were small and had low birth weights, due to growth restriction caused by *MTHFR* dysfunction.^17^^,^^18^ Moreover, several studies reported that variation in *MTHFR C677T* genotype resulted in different pregnancy outcomes. Newborns homozygous for *C677T* have approximately double the risk of adverse birth outcomes (preterm birth: odds ratio OR 1.8, 95% CI 1.0–3.3; low birth weight: odds ratio 1.9, 95% CI 1.0–3.5).^19^ Therefore, future studies should examine whether co-occurrence of *MTHFR C677T* in mother and fetus increases the risk of undesirable pregnancy outcomes.

The importance of birth weight to lifelong health has been a consistent theme during the last few decades. Birth weight is a good surrogate marker of intrauterine growth retardation caused by diverse factors. Low birth weight increases the risk of chronic diseases and adversely affects the health of subsequent generations.^20^ For that reason, a number of studies have used birth weight as a main outcome. In our previous study, we found that birth weights of offspring from mothers with hyperhomocysteinemia (≥15 µmol/L) were significant lower than those of offspring from mothers with normal homocysteine concentrations.^21^ However, that study did not adjust for relevant confounding factors and did not use an adequate statistical approach, such as multilevel modeling for path construction. Folic acid supplementation has been proposed as a treatment of hyperhomocysteinemia. A randomized control trial observed that oral folic supplementation significantly reduced plasma homocysteine level.^22^ Other studies reported that consumption of folic acid attenuated the risk of preterm birth in women with pre-eclampsia.^16^^,^^23^ However, lowering homocysteine level does not decrease disease risk. Hence, future studies should examine the true effect of folic acid supplementation on adverse pregnancy outcomes.

This study had several limitations. First, in a genetic epidemiology study the results of Mendelian randomization may be confounded by genetic variants in linkage disequilibrium with the variant of interest. In addition, unmeasured and residual confounding factors, such as nutritional intake during pregnancy, may have affected the results. A number of factors in the model, including homocysteine and maternal age, are associated with miscarriage and adverse birth outcomes. Because only women who successfully completed pregnancy were included in the present study, there is a possibility of selection bias. Last, due to lack of statistical power, generalizability of the results is problematic—similar previous studies enrolled more than 1000 people. Despite the relative small size of the present study, we obtained valuable results from IV analysis. Future studies should investigate the effect of homocysteine on birth outcomes by considering the genotypes of many people.

A strength of the present study is that it used an innovative study methodology that differed from the designs of previous studies, which were mostly case-control studies.^24^ The present advanced statistical method also allows for inference of causality. In addition, by considering a genetic polymorphism as an instrumental variable, it can assess unmeasurable effects. In conclusion, our results provide more evidence of an adverse relationship between maternal homocysteine level and birth outcomes and suggest that reduction of homocysteine levels during pregnancy could positively affect birth outcomes.
